# Digenic Inheritance of *LAMA4* and *MYH7* Mutations in Patient with Infantile Dilated Cardiomyopathy

**DOI:** 10.3390/medicina55010017

**Published:** 2019-01-15

**Authors:** Atiyeh M Abdallah, S. Justin Carlus, Abdulhadi H Al-Mazroea, Mohammad Alluqmani, Yousef Almohammadi, Zahurul A Bhuiyan, Khalid M Al-Harbi

**Affiliations:** 1 West Midlands Regional Genetics Laboratory, The Birmingham Women’s and Children’s NHS Foundation Trus, Birmingham B15 2TT, UK; 2 Cardiogenetics Unit, Pediatrics Department, College of Medicine, Taibah University, Al-Madinah 30001, Saudi Arabia; justincarlus@gmail.com (S.J.C.); abdalhadi666@yahoo.com (A.H.A.-M.); mallugmani@moh.gov.sa (M.A.); kalharbi@taibahu.edu.sa (K.M.A.-H.); 3 Security Forces Medical Centre, Al-Madinah 30010, Saudi Arabia; almohammadi@msn.com; 4 Unit of Cardiogenetics Research, Division of Genetic Medicine, BT.02. 251, Beaumont 29, 1011 Lausanne, Switzerland; z.a.bhuiyan@chuv.ch

**Keywords:** targeted gene sequencing, dilated cardiomyopathy, digenic, *MYH7*, *LAMA4*, Saudi Arabia

## Abstract

*Background and objectives*: Dilated cardiomyopathy (DCM) is a rare cardiac disease characterised by left ventricular enlargement, reduced left ventricular contractility, and impaired systolic function. Childhood DCM is clinically and genetically heterogenous and associated with mutations in over 100 genes. The aim of this study was to identify novel variations associated with infantile DCM. *Materials and Methods*: Targeted next generation sequencing (NGS) of 181 cardiomyopathy-related genes was performed in three unrelated consanguineous families from Saudi Arabia. Variants were confirmed and their frequency established in 50 known DCM cases and 80 clinically annotated healthy controls. *Results*: The three index cases presented between 7 and 10 months of age with severe DCM. In Family A, there was digenic inheritance of two heterozygous variants: a novel variant in *LAMA4* (c.3925G > A, p.Asp1309Asn) and a known DCM mutation in *MYH7* (c.2770G > A; p.Glu924Lys). The *LAMA4* p.Asp1309Asn variant was predicted to be likely pathogenic according to international guidelines. The other two families had no identifiable potentially deleterious variants. *Conclusions*: Inheritance of two genetic variants may have a synergistic or dose effect to cause severe DCM. We report of a novel p.Asp1309Asn variation associated with DCM. Targeted NGS is useful in the molecular diagnosis of DCM and to guide whole-family management and counselling.

## 1. Introduction

Cardiomyopathy is classified by the European Society of Cardiology into four main classes: dilated cardiomyopathy (DCM), hypertrophic cardiomyopathy (HCM), restrictive cardiomyopathy (RCM), and arrhythmogenic right ventricular cardiomyopathy (ARVC) [1]. A fifth subgroup includes left ventricular (LV) non-compaction and Takotsubo cardiomyopathy [1]. Of these, DCM is a rare cardiac disease characterised by LV enlargement, reduced LV contractility, and impaired systolic function, which can lead to sudden death [2]. Due to a lack of large studies, ethnic differences, and variable DCM diagnostic criteria, the disease prevalence data are inconsistent [3], but prevalences of 1 in 2700 have been reported in the USA [4] and 1 in 7000 in Japan [5]. Familial DCM is present in almost 50% of reported cases, mostly inherited in an autosomal dominant pattern, although some cases reported with X-linked, autosomal recessive and mitochondrial DNA inheritance [6].

The familial pattern of DCM is complicated by incomplete penetrance, high variability in age of onset and disease progression, and high genetic heterogeneity [7]. The incidence of DCM in the US is 13-times greater in infants than in older children and has a male predominance [8]. Similarly, over 50% of presentations were before 14 months of age but with a female predominance in Saudi Arabia [9] and 20% of cases were familial, which is consistent with global estimates of a hereditary aetiology in 20% to 35% of cases [10].

DCM can be secondary to other, reversible disorders such as immunological reactions, toxins, and metabolic disturbances, but idiopathic DCM is often a progressive disease. Genetic testing can identify individuals with hereditary DCM and help pre-symptomatic family members manage disease risk. Cardiomyopathies are associated with >100 known genes [11] including genes encoding for sarcomere, Z-disk, tafazzin, sodium and potassium channel, RNA-binding protein, cytoskeletal, and dystrophin proteins [6]. Therefore, studying the pathogenesis of DCM is highly challenging due to the gene and locus heterogeneity, and is a challenge for diagnostic laboratories.

However, targeted next-generation sequencing (NGS) technology has been used to overcome the limitations of conventional Sanger sequencing. Further, NGS is becoming a more cost-effective method capable of generating large amounts of data for each patient. Targeted NGS and multi-gene panels are now available in clinical diagnostic laboratories with a fast turnaround time (TAT). Compared to whole exome sequencing, targeted sequencing offers value and reduced TATs, an increased coverage depth, and less complicated data processing, making it ideal for routine clinical applications [12,13].

The majority of reported DCM mutations are rare or private mutations [7]. Moreover, with targeted sequencing technology, multiple variants are increasingly reported and are often associated with high disease severity. For example, Haas et al. [7] screened 639 DCM patients for variants in 84 genes known to be associated with cardiomyopathy and reported that 12.8% of the cohort have at least three known variants.

Here we aimed to identify novel mutation(s) and gene(s) responsible for DCM using targeted NGS with an extended gene panel for the coding sequences and flanking regions of 181 genes associated with cardiomyopathy in three unrelated consanguineous Saudi Arabian families. In doing so, we identified digenic *MYH7* and *LAMA4* variants in an affected DCM individual.

## 2. Materials and Methods

### 2.1. Subjects

**Figure 1:**
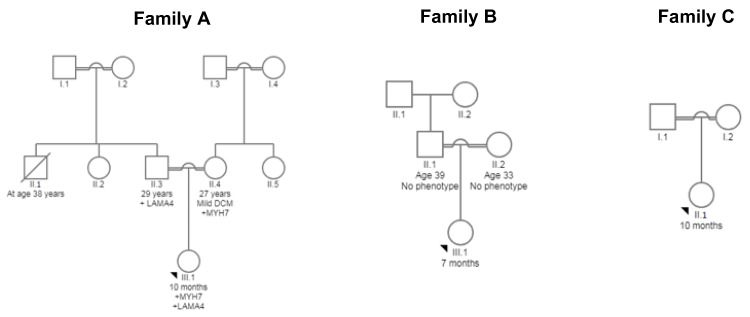
The pedigrees of families with DCM showing the three affected probands. Male family members are indicated with squares; females with circles; deceased individuals are indicated with strikethroughs. The probands are marked with black arrows. The presence of a mutation is indicated by a “+” sign. The number below the symbol represents the age. Trios were investigated by targeted sequencing.

Peripheral blood samples were collected from three trios (nine individuals): three affected DCM probands and their unaffected parents from consanguineous Saudi Arabian families attending the Department of Pediatric Cardiology, MMCH, Al-Madinah, Saudi Arabia (Figure 1) [14]. Family A and B reported a family history of sudden death from “heart disease”. All patients resided in Al-Madinah, western Saudi Arabia. Probands were between 7 and 11 months of age at the time of presentation. Disease management and clinical investigations were commenced on admission, which included diagnosis of early-onset DCM by a certified cardiologist. Clinical investigations included X-ray, ECG, and echocardiography. Other cardiac or systemic causes of DCM including coronary and valvular diseases were ruled out.

The study was conducted in accordance with the ethical standards of the Taibah University Ethical Research Committee and the Ethical Review Board of Madina Maternity and Children Hospital (MMCH). The authors applied the World Medical Association Declaration of Helsinki. Parents provided written, informed consent.

### 2.2. Targeted Sequencing

Genomic DNA was extracted from peripheral blood using the QIAamp DNA Blood Kit (Qiagen, Hilden, Germany). An in-house custom gene panel covering the coding exons and exon–intron boundaries of 181 genes either confirmed or presumed to be involved in cardiomyopathy was used as previously described [11,15]. A gene list was curated using the search terms “Hypertrophic cardiomyopathy, HCM, dilated cardiomyopathy, and DCM from PubMed, Online Mendelian Inheritance in Man (OMIM) and peer-reviewed reports (Supplementary Data Table S1). The total length of sequenced DNA was 1,108,523 bp involving 3852 targets. All annotated coding regions were extracted from hg19 from ENSEMBL (http://www.ensembl.org) and the UCSC genome browser (http://genome.ucsc.edu).

The library was enriched using a custom Nimblegen panel and sequenced as 2 × 250 bp paired-end reads on an Illumina Hiseq2500 (Illumina, San Diego, CA, USA). Bioinformatic analysis was performed as previously described [16]. ConSurf analysis was conducted using the HMMER homology search algorithm with a single iteration against the UNIREF-90 database [17]. Target sequences were aligned with the top 100 sequences in UNIREF-90.

Sanger sequencing was performed to confirm the presence of variants. Primer sequences were designed using Primer3. The *MYH7* forward primer was 5′-AGGACCTTACCCCCTGAACA-3′, and the reverse primer was 5′-GCCTGGGTCAAGGTCAGTATG-3′. The *LAMA4* forward primer was 5′-CGAACAGGAGAAAGCCACAC-3′, and the reverse primer was 5′-CCCTTCTTGGTGTACCATCACA-3′. PCR amplicons were sequenced using BigDye chemistry on the ABI3500 DNA analyser (Applied Biosystems, Foster City, CA, USA). Data were analysed using Auto Assembler Software (Applied Biosystems).

### 2.3. Variant Classification

Variant pathogenicity was assessed and classified as either pathogenic, likely pathogenic, variant of unknown significance, likely benign, or benign—based on the American College of Medical Genetics (ACMG) [18] and Association for Clinical Genomic Science (ACGS)-UK guidelines [19].

### 2.4. Additional Screening of the DCM Cohort and Controls

The identified variant was screened in 80 clinically-annotated controls and 50 DCM cases to evaluate its frequency in the Saudi Arabian general population as well as in individuals with DCM. This control cohort is described in detail elsewhere [15].

## 3. Results

### 3.1. Clinical Data

The three probands developed DCM at under one year of age and presented to the Paediatric Cardiology Unit, MMCH, Saudi Arabia. Two patients were female and one was male. The affected probands all had clinically severe DCM.

**Figure 2:**
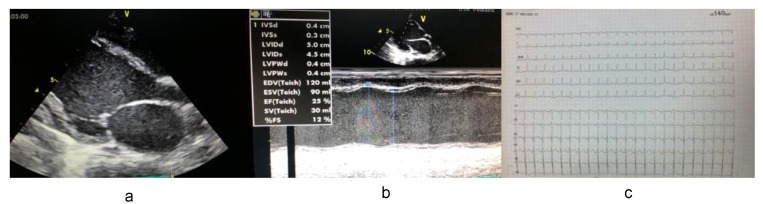
(**a**,**b**) Echocardiographic recording from the index patient in Family A showing severe DCM with marked systolic and diastolic dysfunction and enlarged heart chambers; (**c**) Electrocardiography of the proband demonstrated normal sinus tachycardia, a heart rate of 140 beats/min, a normal axis, and a widened QRS complex that may indicate a left bundle branch block.

In Family A, the proband presented at 10 months of age with symptoms and signs of decompensated heart failure. Echocardiography revealed a severe form of DCM with marked systolic and diastolic dysfunction, left ventricle end-diastolic diameter (LVED) of 120 cm, left ventricular end systolic diameter (LVES) of 0.4 cm, a low left ventricular ejection fraction (LVEF) of 25%, and left ventricular posterior wall thickness of 0.4 cm (Figure 2a,b). ECGs revealed normal sinus tachycardia, a heart rate of 140 beats/min, a normal axis, and a widened QRS complex (>110 ms) possibly indicating a left bundle branch block (Figure 2c). For families B and C, probands were also diagnosed with DCM. In Family B, the proband presented at 7 months of age with symptoms and signs of heart failure. Echocardiography revealed DCM with marked systolic and diastolic dysfunction, enlarged left ventricle (LVED 117 cm, LVES 0.35 cm), a low LVEF of 22%, and left ventricular posterior wall thickness of 0.3 cm. ECGs revealed normal sinus tachycardia, a heart rate of 138 beats/min, a normal axis, and a widened QRS complex (>110 ms). In Family C, the proband presented at 10 months of age with symptoms and signs of heart failure. Echocardiography revealed DCM with marked systolic and diastolic dysfunction, enlarged left ventricle (LVED 110 cm, LVES 0.3 cm), a low LVEF of 19%, and left ventricular posterior wall thickness of 0.27 cm. ECGs revealed normal sinus tachycardia, a heart rate of 140 beats/min, a normal axis, and a widened QRS complex (>105 ms). Symptoms of metabolic disorders were completely absent in all probands.

### 3.2. Identification of Variants

Targeted NGS was performed on the three affected DCM probands and their unaffected parents, hypothesising that members of an affected family would have the same genotype but that severely affected individuals might have multiple variants in the same or other genes. Therefore, we used targeted sequencing of a multi-gene panel to try and cover as many putative DCM-related genes as possible. Assuming an autosomal dominant, Mendelian disease model, and under the assumption that rare heterozygous mutations may be causative of DCM, filters were applied to identify variants meeting these criteria. A read depth of 30 was considered a correct read with a sufficiently covered region based on a Q30 = 99.9% chance that the base was correctly called.

**Figure 3:**
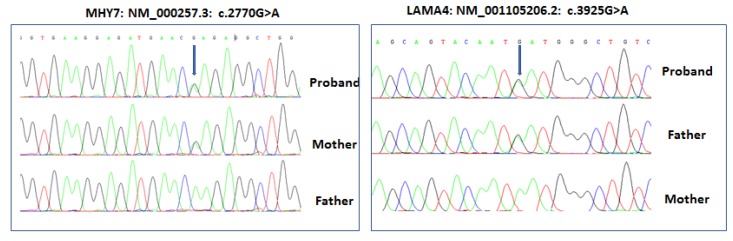
Sanger sequencing of the Family A trio. The *MYH7* p.E924K mutation was present in the heterozygous state in the case and mildly affected mother. The *LAMA4* p.Asp1309Asn variant was present in the heterozygous state in the case and unaffected father. Variants are indicated with arrows.

Variants were confirmed by Sanger sequencing (Figure 3). The identified variants were absent in 50 paediatric DCM cases and 80 controls [15]. Targeted sequencing for the other two affected probands in Family B and Family C (Figure 1) did not identify any likely pathogenic or pathogenic variants in the 181 genes tested in this panel.

### 3.3. Variant Classification

The affected child in Family A had two heterozygous variants in two different genes (Figure 1 and Figure 3). The first variant was NM_000257.3: c.2770G > A; p.Glu924Lys in *MYH7* (ClinVar Accession VCV000014092.2)—a variant not previously reported in public control databases (the Genome Aggregation Database (gnomAD, http://gnomad.broadinstitute.org/) and the Greater Middle East Variome Project database (GME Variome http://igm.ucsd.edu/gme/)) [20] but reported in ClinVar and the Atlas of Cardiac Genetic Variation (https://www.cardiodb.org/acgv/index.php) as associated with HCM. However, the pathogenicity of this variant in DCM has not been clinically demonstrated.

**Figure 4:**
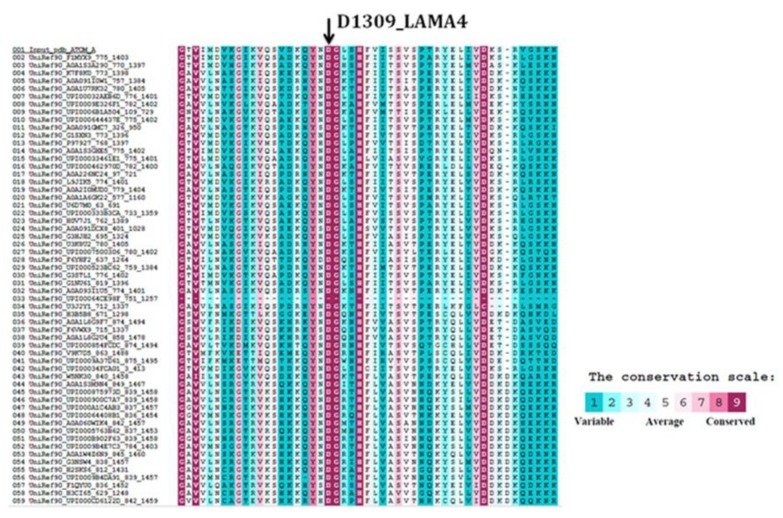
Location and conservation of the mutated amino acids. The *LAMA4* p.Asp1309Asn variant affects a highly conserved leucine residue that is conserved in all analysed species.

The second missense variant was a novel variant in *LAMA4* (NM_001105206.2: c.3925G > A, p.Asp1309Asn, which was not present in any control databases including gnomAD, GME Variome, and dbSNP or the Atlas of Cardiac Genetic Variation, and in disease databases such as ClinVar and the Atlas of Cardiac Genetic Variation. This suggests that it may be a “private” variant in this family. The variant changes an acidic aspartic acid to a neutral polar uncharged asparagine in the laminin G-like 3 domain. ConSurf analysis showed that the aspartic acid at codon 1309 had a conservation score of 9, meaning that the residue is highly evolutionarily conserved (Figure 4). In silico analysis using SIFT, MutationTaster, and Polyphen-2 predicted that the *LAMA4* p.Asp1309Asn variant was “damaging”. Based on the above data and following the ACMG and ACGS variant classification guidelines, we classified the novel *LAMA4* p.Asp1309Asn variant as “likely pathogenic” based on three evidences (PM1, PM2, PP3, PP4). However, automated classification software, such as VarSome (https://varsome.com), assigned a classification of “variant of unknown significance (VUS) based on two evidences (PM2 and PP3). The *MYH7* p.Glu924Lys variant is well described and classified as pathogenic in ClinVar and the Atlas of Cardiac Genetic Variation.

## 4. Discussion

Here we used targeted sequencing of 181 cardiomyopathy-related genes to identify the underlying genetic causes in three unrelated consanguineous families with DCM. We identified variants in two genes in family A. The first variant is p.Glu924Lys in *MYH7*, a well-documented missense variant that has been classified as pathogenic in its association with HCM and previously reported in the Atlas of Cardiac Genetic Variation and ClinVar. The second variant is a novel, missense variant in *LAMA4* (p.Asp1309Asn), not reported in control databases including gnomAD, GME Variome, and dbSNP. Two other variants within 10 amino acids either side of p.Asp1309Asn were reported in gnomAD as singletons (less than 1 in 10,000), suggesting that this locus may be a mutational hotspot.

The family consisted of first-degree cousin parent. The mother carried the *MYH7* p.Glu924Lys mutation and had mild DCM at age 27. The father carried the *LAMA4* p.Asp1309Asn variant and did not have echocardiographic manifestations of DCM at age 29. The proband phenotype was severe infantile DCM. These observations highlight the importance of genetic and echocardiographic screening of family members of patients with DCM.

DCM is a genetically heterogeneous condition, associated with different genes including those with gene products involved in cytoskeletal, nucleoskeletal, mitochondrial, and calcium pathways. Many reported mutations in these pathways are rare variants that are unique to specific families. The most frequent DCM-associated gene is *TTN*, mutated in 18% and 25% of sporadic and familial DCM cases, respectively [7] compared to mutations in *MYH7* and *MYBPC3* accounting for 83% of HCM cases [13]. Some DCM-related mutations DCM or HCM disease at different ages of onset, even within the same family [21].

The *MYH7* p.Glu924Lys variant was first reported by Watkins et al. in 1992 [22] in two members of the same family affected by HCM out of 25 unrelated families screened. Interestingly, they found no disease-related or sudden death in this family, whereas disease-related deaths and sudden deaths were much more common in families carrying the *MYH7* Arg403Gln variant. The p.E924K variant may, therefore, produce a more subtle phenotype. Morner et al. [23,24] reported a case in a Swedish patient digenic for *MYH7* p.E924K and *MYBPC3* Val896Met mutations, the carriers of the *MYBPC3* variant apparently having late-onset HCM. *MYH7* plays an important role in sarcomere function—the contracting unit of cardiomyocytes, and forms a thick filament core with MYBPC3 in cardiomyocytes [6]. *MYH7* mutations have also been reported in other cardiomyopathies such as non-compaction cardiomyopathy and restrictive cardiomyopathy [25]. Therefore, the role that *MYH7* mutations play in the onset of different cardiomyopathy phenotypes is unclear. It has been proposed that modifier genes and the environment play a role in the final disease phenotype [26].

The *LAMA4* p.Asp1309Asn variant has not been reported. Laminins are important glycoproteins for the basement membrane extracellular matrix (ECM) that provide structural stability to cells and have signalling functions [27]. *LAMA4* encodes the laminin α4 chain [27]. *LAMA4* is implicated in neuromuscular diseases [28], and *LAMA4* mutations have been reported in DCM and HCM patients. Knoll et al. [29] screened 180 severe DCM patients and detected two *LAMA4* mutations. Functional studies in zebrafish have shown that *LAMA4* knockdown causes defects in endothelial cells, heart dysfunction, and haemorrhage in 35% of embryos. In addition, in mice, the *LAMA4* transcript is mostly expressed in cardiac and skeletal muscle and the lung [30]. Mice lacking *LAMA4* develop cardiomyopathy and have an increased frequency of sudden death upon stress; electron microscopy of these mice revealed malformed blood vessels and micro-circulation abnormalities [31]. Moreover, patients carrying a *LAMA4* Pro943Leu mutation have a significantly reduced extracellular matrix (ECM) in cardiomyocytes [29]. These findings support the importance of *LAMA4* as a structural and signalling molecule in cardiomyocytes, and may indicate the modifier role that missense variations in *LAMA4* play in the disease.

Digenic heterozygosity has been described in some DCM cases and is often associated with a severe presentation of DCM. Moller et al. [32] reported an index case with digenic variants in *MYH7* (L1038P) and *MYBPC3* (R326Q), both encoding sarcomeric proteins that are likely to affect its structure when mutated. Petropoulou et al. [33] reported a family severely affected by DCM and who had two digenic variations in *MYH7* (Asp955Asn) and *TNNT2* (Asn83His), both sarcomeric genes. Here we reported heterozygous variants in genes that play roles in two different cardiomyocyte components; *MYH7*—part of the sarcomere, and *LAMA4*—part of the ECM/signalling component. To our knowledge, this is the first description of digenic mutations in *MYH7* and *LAMA4*. The mutations were inherited from the parents, the mother carrying a *MYH7* mutation and with mild DCM, and a father carrying the *LAMA4* variation but with a normal heart at age 29. It is unclear whether the latter mutation can cause DCM on its own or if it plays a modifier role in the disease, however, long-term follow-up might clarify the association. Given the severe DCM phenotype in the proband at age 10 months and the presence of two different mutated genes in two different cardiomyocyte components, we believe that the *LAMA4* p.Asp1309Asn variation found here may cause a mild defect in *LAMA4* function that only manifests in combination with the digenic inheritance of the *MYH7* p.Asp1309Asn mutation.

Our study is limited by a lack of functional data on the *LAMA4* p.Asp1309Asn variant. The identified variants were completely absent in 80 clinically annotated controls and 50 sporadic paediatric DCM cases. We propose that the digenic inheritance of these two variants caused the severe infantile DCM phenotype in this family. In addition, as a trio study, whole exome sequencing would explore any additional genetic variations in these families and would provide a better understanding of the disease mechanism. Another limitation of our study is that a SNP array was not performed. Because families are consanguineous, it is possible that an additional autosomal recessive disease or epigenetic disorder could be contributing to the proband phenotypes.

## 5. Conclusions

In conclusion, this study identified a severe case of DCM with digenic inheritance of two variants in *MYH7* and *LAMA4* detected by a 181-gene NGS panel. These results highlight the usefulness of using extended and large gene panels that include all cardiomyopathy-related genes to identify causative variants in DCM. However, as we could not find any pathogenic variants in the other two families analysed, these targeted panels need further expansion or, alternatively, whole exome sequencing technology is a good alternative option once the technology becomes affordable and the analysis becomes manageable for diagnostic laboratories. Identifying genetic variations in DCM will help to understand the underlying pathogenetic mechanisms, guide clinical management and genetic counselling, and direct personalised treatment.

## Supplementary Materials

The following are available online at http://www.mdpi.com/1010-660X/55/1/17/s1.
