# Supplementary material for: Digenic Inheritance of LAMA4 and MYH7 Mutations in Patient with Infantile Dilated Cardiomyopathy
Source: Medicina (Kaunas). 2019 Jan 15;55(1):17. doi: 10.3390/medicina55010017 (PMC6359299; doi:10.3390/medicina55010017)
Supplement: Supplementary file 1 [file medicina-55-00017-s001.pdf]

Table S1: List of 181 Genes screenen.

ABCC9, ACADVL, ACTA1, ACTC1, ACTN2, AKAP9, ALG10, AMPD1, ANK2, ANO5, B3GALNT2, B4GAT1, BAG3, BIN1, BMPR2, CACNA1C, CACNA1D, CACNA2D1, CACNB2, CALM1, CALM2, CALR3, CAPN3, CASQ2, CAV3, CCDC78, CFL2, CHKB, CLIC2, CNBP, CNTN1, COL12A1, COL6A1, COL6A2, COL6A3, COL9A3, CRYAB, CSRP3, CTNNA3, DAG1, DES, DMD, DMPK, DNAJB6, DNAJC19, DNM2, DPP6, DSC2, DSG2, DSP, DTNA, DYSF, EMD, EYA4, FHL1, FKRP, FKTN, FLNC, GATAD1, GJA1, GJA5, GLA, GMPPB, GNAI2, GNE, GPD1L, HCN4, HNRNPDL, HRAS, ISCU, ISPD, ITGA7, JPH2, JUP, KBTBD13, KCNA5, KCND3, KCNE1, KCNE2, KCNE3, KCNH2, KCNJ2, KCNJ5, KCNQ1, KLHL40, KLHL41, LAMA2, LAMA4, LAMP2, LARGE, LDB3, LMNA, LMOD3, MEGF10, MIB1, MTM1, MYBPC1, MYBPC3, MYF6, MYH2, MYH6, MYH7, MYL2, MYL3, MYLK2, MYOT, MYOZ2, MYPN, NEB, NEXN, NKX2-5, NPPA, NUP155, PABPN1, PKP2, PLEC, PLN, PNPLA2, POLG, POMGNT1, POMGNT2, POMK, POMT1, POMT2, PRDM16, PRKAG2, PSEN1, PSEN2, PTRF, RAF1, RBM20, RRM2B, RYR1, RYR2, SCN10A, SCN1B, SCN2B, SCN3B, SCN4B, SCN5A, SCO2, SDHA, SEPN1, SGCA, SGCB, SGCD, SGCG, SLC22A5, SLC25A4, SMCHD1, SNTA1, SPEG, STAC3, SYNE1, SYNE2, TAZ, TBX20, TCAP, TGFB3, TIA1, TMEM43, TMEM5, TMPO, TNNC1, TNNI2, TNNI3, TNNT1, TNNT2, TNPO3, TPM1, TPM2, TPM3, TRAPPC11, TRDN, TRIM32, TRPM4, TTN, TTR, VCL, VCP, VMA21.
